# CutFEM forward modeling for EEG source analysis

**DOI:** 10.3389/fnhum.2023.1216758

**Published:** 2023-08-22

**Authors:** Tim Erdbrügger, Andreas Westhoff, Malte Höltershinken, Jan-Ole Radecke, Yvonne Buschermöhle, Alena Buyx, Fabrice Wallois, Sampsa Pursiainen, Joachim Gross, Rebekka Lencer, Christian Engwer, Carsten Wolters

**Affiliations:** ^1^Institute for Biomagnetism and Biosignalanalysis, University of Münster, Münster, Germany; ^2^Institute for Analysis and Numerics, University of Münster, Münster, Germany; ^3^Department of Psychiatry and Psychotherapy, University of Lübeck, Lübeck, Germany; ^4^Center of Brain, Behaviour and Metabolism, University of Lübeck, Lübeck, Germany; ^5^Otto Creutzfeldt Center for Cognitive and Behavioral Neuroscience, University of Münster, Münster, Germany; ^6^Institute of History and Ethics in Medicine, Technical University of Munich, Munich, Germany; ^7^Institut National de la Santé et de la Recherche Médicale, University of Picardie Jules Verne, Amiens, France; ^8^Computing Sciences Unit, Faculty of Information Technology and Communication Sciences, Tampere University, Tampere, Finland; ^9^Institute for Translational Psychiatry, University of Münster, Münster, Germany

**Keywords:** EEG forward problem, realistic head modeling, volume conductor modeling, unfitted FEM, level set, finite element method

## Abstract

**Introduction:**

Source analysis of Electroencephalography (EEG) data requires the computation of the scalp potential induced by current sources in the brain. This so-called EEG forward problem is based on an accurate estimation of the volume conduction effects in the human head, represented by a partial differential equation which can be solved using the finite element method (FEM). FEM offers flexibility when modeling anisotropic tissue conductivities but requires a volumetric discretization, a mesh, of the head domain. Structured hexahedral meshes are easy to create in an automatic fashion, while tetrahedral meshes are better suited to model curved geometries. Tetrahedral meshes, thus, offer better accuracy but are more difficult to create.

**Methods:**

We introduce CutFEM for EEG forward simulations to integrate the strengths of hexahedra and tetrahedra. It belongs to the family of unfitted finite element methods, decoupling mesh and geometry representation. Following a description of the method, we will employ CutFEM in both controlled spherical scenarios and the reconstruction of somatosensory-evoked potentials.

**Results:**

CutFEM outperforms competing FEM approaches with regard to numerical accuracy, memory consumption, and computational speed while being able to mesh arbitrarily touching compartments.

**Discussion:**

CutFEM balances numerical accuracy, computational efficiency, and a smooth approximation of complex geometries that has previously not been available in FEM-based EEG forward modeling.

## 1. Introduction

Electroencephalography (EEG) is a widely used tool for the assessment of neural activity in the human brain (Brette and Destexhe, 2012). To estimate the area of the brain responsible for the measured data, one has to simulate the electric potential as induced by hypothetical current sources in the brain, i.e., the EEG forward problem has to be solved. While quasi-analytical solutions to the differential equation underlying the forward problem exist, these are only available in simplified geometries such as the multi-layer sphere model (De Munck and Peters, 1993). One, thus, requires numerical methods to incorporate accurate representations of the head's shape and volume conduction properties. Popular approaches are the boundary element method (BEM) (Mosher et al., 1999; Gramfort et al., 2011; Makarov et al., 2020), finite difference method (FDM) (Song et al., 2015; Cuartas Morales et al., 2019), and the finite element method (FEM) (Zhang et al., 2004; Vallaghé and Papadopoulo, 2010; Medani et al., 2015; Acar et al., 2016; Azizollahi et al., 2018). Here, we will focus on the FEM due to its flexibility in modeling complex geometries with inhomogeneous and anisotropic compartments (Schimpf et al., 2002; Van Uitert et al., 2004; Wolters et al., 2007; Bangera et al., 2010; Nüßing et al., 2016; Beltrachini, 2018; He et al., 2020; Vermaas et al., 2020). Efficient solvers and the transfer matrix approach (Wolters et al., 2004; Lew et al., 2009) allow significantly reduced computational costs.

When employing FEM, one usually chooses between either a hexahedral or tetrahedral discretization of the head. Both choices come with their own strengths and limitations. The mesh creation requires a classification of the MRI into tissue types. This segmentation data often come in the form of binary maps with voxels of approximately 1mm resolution, allowing for quick and simple hexahedral mesh generation. However, as head tissue surfaces are smooth, approximating them with regular hexahedra is bound to be inaccurate. While the methods for geometry adaptation exist (Wolters et al., 2007), the resulting meshes still have an (reduced) angular pattern. Furthermore, when applying a standard continuous Galerkin FE scheme, areas with very thin compartments may suffer from leakage effects where current can bypass the insulating effects of the skull (Sonntag et al., 2013). To alleviate this, flux-based methods, such as the discontinuous Galerkin method, offer a robust alternative (Engwer et al., 2017). These, however, severely increase the number of degrees of freedom (DOF) and thus necessary for computational effort.

Surface-based tetrahedral FEM approaches, on the other hand, are able to accurately model the curvature of smooth tissue surfaces. Creating high quality tetrahedra, e.g., ones fulfilling a delaunay criterion, requires tissue surface representations in the form of triangulations first. These triangulations have to be free of self-intersections and are often nested, usually leading to modeling inaccuracies such as neglecting skull holes or an artificial separation of gray matter and skull. Therefore, we will not discuss surface-based tetrahedral FEM approaches throughout this study.

In the study by Rice et al. (2013), the impact of prone vs. supine subject positioning on EEG amplitudes was investigated. In the small group study, average differences of up to 80% were found. These were accompanied by differences in MRI-based CSF-thickness estimation of up to 30% underlining the importance of correctly modeling CSF-thickness and areas of contact between the skull and brain surfaces.

Recently, an unfitted discontinuous Galerkin method (UDG) (Bastian and Engwer, 2009) was introduced to solve the EEG forward problem (Nüßing et al., 2016). Rather than working with mesh elements that are tailored to the geometry, it uses a background mesh which is cut by level set functions, each representing a tissue surface. It was shown to outperform the accuracy of a discontinuous Galerkin approach on a hexahedral mesh while not being limited by the assumptions necessary to create tetrahedral meshes.

Extending the ideas of the UDG method, this study introduces a multi-compartment formulation of the CutFEM (Burman et al., 2015) for EEG source analysis. Compared with UDG, it operates on a simpler trial function space and adds a ghost penalty based on the study by Burman (2010). The ghost penalty couples small mesh elements to their neighbors to improve the conditioning of the method.

This study is structured as follows. After introducing the theory behind CutFEM, three successively more realistic scenarios are tested. These scenarios include a multi-layer sphere model, followed by realistic brain tissues embedded in spherical skull and scalp compartments. Finally, a fully realistic five-compartment head model is used for source analysis of the P20/N20 component of measured somatosensory evoked potentials (SEP). Comparison results from different FEM and meshing approaches will be considered throughout the scenarios.

## 2. Methods

### 2.1. A cut finite element method

Deviating from classical, fitted FEM-approaches, where the mesh cells resolve tissue boundaries, CutFEM uses a level set-based representation of domain surfaces. Let Ω = ⋃_*i*_Ω_*i*_ be the head domain divided into *m* disjunct open subdomains, e.g., the gray matter, white matter, CSF, skull, and skin. The level set function for compartment *i* is then defined as follows:


$$\Phi_{i}(x)\left\{ \begin{array}{l} <\;0,\text{ if }x\in \Omega_{\text{i}}\\ =\;0,\text{ if }x\in \partial \;\Omega_{\text{i}}\\ >\;0,\text{ else} \end{array}\right.$$

and $L_{i}=\left\{x\in \Omega :\Phi_{i}\left(x\right)=0\right\}$ denotes its (zero) level set. We proceed by defining a background domain $\hat{\Omega }\subset {\mathbb{R}}^{3}$ covering the head domain Ω. This background is, then tesselated, yielding a regular hexahedral mesh $T\left(\hat{\Omega }\right)$, the fundamental or background mesh. Taking on the level set representation, submeshes $T_{h}^{i}\subset T_{h}\left(\hat{\Omega }\right)$ are created from the background mesh, containing all cells that have at least partial support within the respective subdomain Ω_*i*_. This results in an overlap of submeshes at compartment interfaces. For each submesh, we define a conforming ℚ_1_ space $V_{h}^{i}$. Thus, up to this point, each submesh is treated the way a conforming Galerkin method would treat the entire mesh.

The difference, then, lies in restricting the trial and test functions to their respective compartment, effectively cutting them off at the boundary and giving rise to the name CutFEM. A fundamental mesh cell intersected by a level set $L_{i}$ is called a cut cell. Their respective fundamental cells are contained in multiple compartments and thus have more DOF. On the other hand, compared with classical conforming discretizations, a coarser mesh resolution can be chosen, as the mesh does not have to follow small geometric features. As the trial functions are only continuous on their respective compartment and cut off at the boundary, using them to approximate the electric potential requires internal coupling conditions at the tissue interfaces. We define the internal skeleton as the union of all subdomain interfaces.


(1) $$\Gamma =\cup \{{\bar{\Omega }}_{i}\cap {\bar{\Omega }}_{j}:\;i\neq j,\;\mu_{d-1}({\bar{\Omega }}_{i}\cap {\bar{\Omega }}_{j})>0\}.$$

μ_*d*−1_ is the d-1 dimensional measure in d-dimensional space. For two sets, *E, F* sharing both a common interface (an element of Γ) and a possibly discontinuous function *u* operating on them we can define a scalar- or vector-valued jump operator as ⟦*u⟧*: = *u*|_*E*_·*n*_*E*_+*u*|_*F*_·*n*_*F*_ with *n*_*E*_, *n*_*F*_ the outer unit normal of the respective set. Additionally, a (skew-)weighted average can be stated as follows:


(2) $$\left\{u\right\}\;=\omega_{E}u{|}_{E}+\omega_{F}u{|}_{F}$$

(3) $$\left\{u\right\}{\;}^{*}=\omega_{F}u{|}_{E}+\omega_{E}u{|}_{F}.$$

with $\omega_{E}=\frac{\delta_{E}}{\delta_{E}+\delta_{F}}$, $\delta_{E}=n_{E}^{t}\sigma_{E}n_{E}.$ Here, σ_*E*_ refers to the symmetric 3 × 3, positive definite electric conductivity tensor on *E*. Notably, ⟦*uv⟧* = ⟦*u⟧*{*v*}^*^+{*u*}⟦*v⟧*. The purpose of these definitions will become clear when deriving the weak formulation for our forward model.

Typically, the EEG forward problem for the electric potential *u* induced by a neural source term *f* is derived from the quasi-static formulation of Maxwell's equations (Brette and Destexhe, 2012).


(4) $$\nabla \;\cdot \;\sigma \;\nabla u=f,\text{ in }\underset{i}{\cup }\Omega_{i}$$

(5) $$\langle \sigma \;\nabla u,n\rangle \;=\;0,\text{ on }\partial \;\bar{\Omega }$$

And in addition we require continuity of the electric potential and the electric current


(6) 〚u〛=0,  on Γ

(7) 〚σ ∇u〛=0,  on Γ.

As trial and test space, we employ *V*_*h*_ as direct sum of all $V_{h}^{i}$.

The weak formulation can be obtained by multiplying with a test function, integrating and applying subdomain-wise integration by parts. This yields:


$$\sum_{i}\left(\int_{\Omega_{i}}\text{​}\sigma \nabla u_{h}^{i}\nabla v_{h}^{i}dx\right)-\int_{\Gamma }\text{​}\{\sigma \nabla u_{h}\}〚v_{h}〛dS=-\sum_{i}\left(\int_{\Omega_{i}}\text{​}fv_{h}^{i}dx\right),$$

where the jump formula for a product of two functions as well as (7) were used. $u_{h}^{i}$ is the restriction of *u*_*h*_∈*V* to *V*_*i*_. A symmetry term $\pm \underset{\Gamma }{\int }\left\{\sigma \nabla v_{h}\right\}⟦u_{h}⟧dS$ is added to end up with either a symmetric or non-symmetric bilinearform.

To incorporate (6), a Nitsche penalty term (Nitsche, 1971) is added that weakly couples the domains. Asymptotically, it enforces continuity of the electric potential over tissue boundaries and ensures the coercivity necessary for the methods' convergence (Burman et al., 2015):


(8) $$P_{\gamma }(u,v)=\gamma \nu_{k}\int_{\Gamma }\frac{\hat{\sigma }}{\hat{h}}\;〚u_{h}〛〚v_{h}〛dS.$$

Here, ν_*k*_, ĥ, and $\hat{\sigma }$ are scaling parameters based on the ratio of cut cell area on each interfaces' side, dimension, degree of trial functions used, and conductivity. See Di Pietro and Ern (2011) for a further discussion. γ is a free parameter to be discussed later.

A challenge is the shape of the cut-cells. Distorted or sliver-like snippets with very small volumes lead to very small entries in the stiffness matrix, deteriorating the conditioning of the forward problem. To alleviate this, a ghost penalty (Burman, 2010) term is used, which takes place on the interfaces of all the fundamental mesh cells cut by a level set. Let


(9) $$\hat{\Gamma }=\cup \{\partial E_{i}:E_{i}\in T_{h},E_{i}\cap \Gamma \neq \emptyset \}.$$

Note the difference between Γ and $\hat{\Gamma }$. Γ operates on compartment interfaces, $\hat{\Gamma }$ on faces of the fundamental mesh. The ghost penalty is then defined as follows:


(10) $$a^{G}(u_{h},v_{h})=\gamma_{G}\int_{\hat{\Gamma }}\hat{h}\;〚\sigma \nabla u_{h}〛\;〚\nabla v_{h}〛\;dS,$$

where γ_*G*_ is again a free parameter, usually a couple orders of magnitude smaller than γ. Penalizing the jump in the gradient ensures that trial functions which are only active on small snippets cannot deviate too strongly from the solution in neighboring cells. When using higher order trial functions, higher order derivatives are no longer zero and have to be penalized as well. Notably, by adding a ghost penalty, the method is no longer fully consistent with the original problem. However, due to the size of γ_*G*_, the effect on the overall result is negligible. The weak CutFEM EEG-forward problem can now be stated as finding the electric potential *u*_*h*_∈*V*_*h*_ such that


(11) $$a(u_{h},v_{h})+a_{n/s}^{N}(u_{h},v_{h})+a^{G}(u_{h},v_{h})=l(v_{h})\;\forall v_{h}\in V_{h},$$

with


$$\begin{array}{l} a(u_{h},v_{h})=\sum_{i}\int_{\Omega_{i}}\sigma \nabla u_{h}^{i}\nabla v_{h}^{i}dx,\\ \;l(v_{h})=-\sum_{i}\int_{\Omega_{i}}fv_{h}^{i}dx \end{array}$$

and


$$\begin{array}{l} a_{n/s}^{N}(u_{h},v_{h}):=-\;\int_{\Gamma }\left\{\sigma \nabla u_{h}\right\}〚v_{h}〛\pm \int_{\Gamma }\left\{\sigma \nabla v_{h}\right\}〚u_{h}〛dS\\ \;+\gamma \nu_{k}\int_{\Gamma }\frac{\hat{\sigma }}{\hat{h}}〚u_{h}〛〚v_{h}〛dS. \end{array}$$

In the following, we will refer to these two variants as NWIPG/SWIPG, short for the non-symmetric/symmetric weighted interior penalty Galerkin method.

In the study by Oden et al. (1998) and Guzmán and Rivière (2009), it was shown that the non-symmetric DG-methods may result in a sub-optimal convergence rate in the L2-norm (full convergence in H1), a result that also extends to CutFEM (Burman and Hansbo, 2012). However, while SWIPG is coercive only if γ is chosen sufficiently large (Burman and Hansbo, 2012), NWIPG does not have such a limitation. Therefore, we will employ the NWIPG method throughout this study due to its stability with regard to the selection of γ.

#### 2.1.1. Integration over the cut domains

Fundamental cells that are cut by level sets, the cut cells; can be integrated over by employing a topology preserving marching cubes algorithm (TPMC) (Engwer and Nüßing, 2017). The initial cell is divided into a set of snippets, each completely contained within one subdomain. These snippets are of a simple geometry and therefore easy to integrate over. Thus, integrals over the fundamental cell or subdomain boundaries are replaced by integrals over the snippets or their boundaries. The trial functions are effectively cut off at the compartment boundaries.

**Figure 1:**
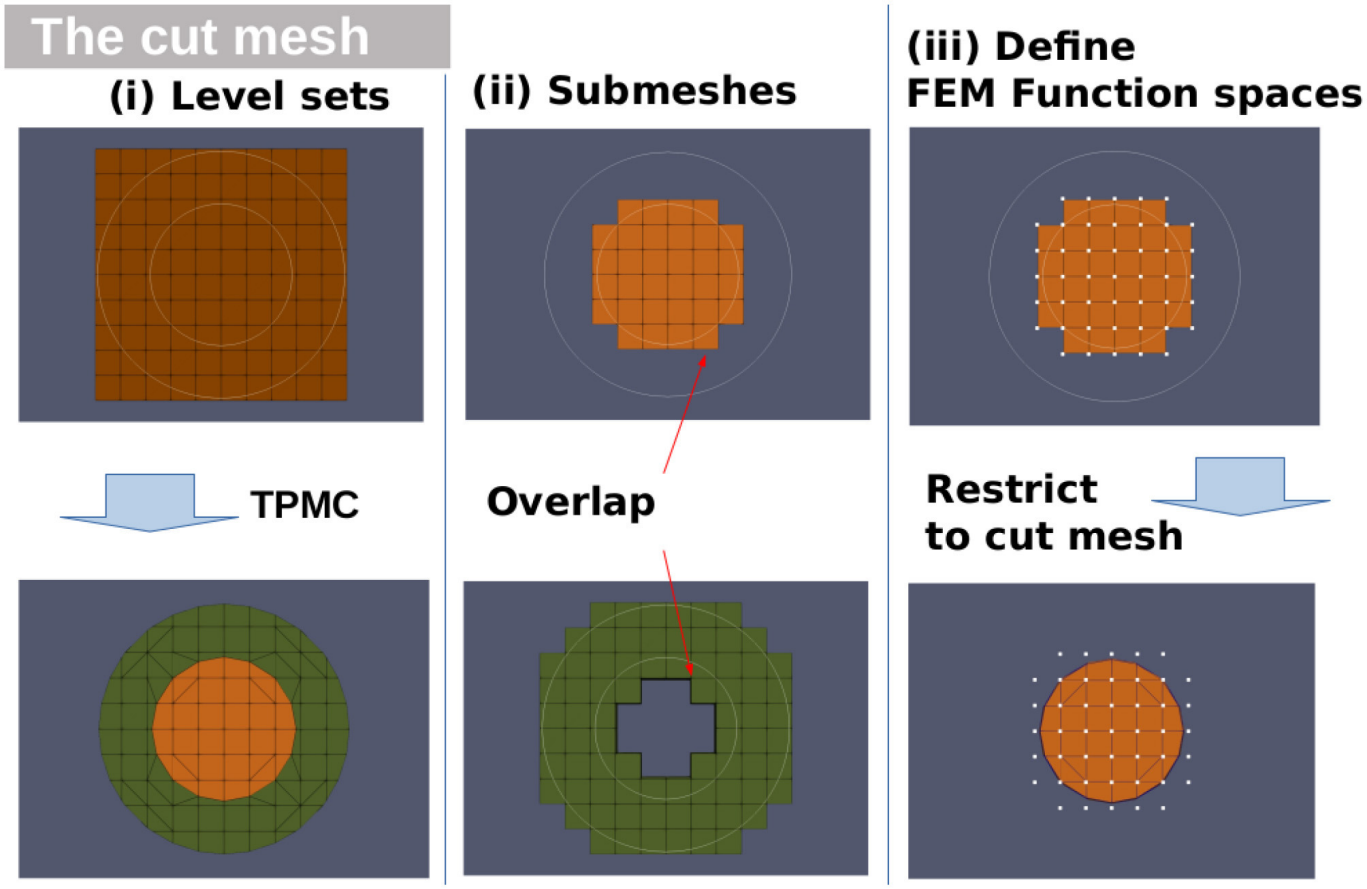
Level sets over fundamental mesh and TPMC reconstruction. **Left**: Fundamental mesh with two spherical level sets, topology preserving marching cubes reconstruction. **Center**: Overlapping submeshes for the two compartments enclosed by the level sets. **Right**: Trial function space for the inner compartment with white dots representing degrees of freedom, cut area that the DOF are restricted to.

See Figure 1 for an overview of the reconstruction steps. Notably, the trial functions are coupled to their respective submesh, not to the TPMC reconstruction of the domain. The latter only determines the area over which the functions are integrated.

Starting on the fundamental mesh, the algorithm is applied once per level set. Each following iteration is applied on the cut cells of the previous iteration, i.e., first the fundamental mesh is cut, then the resulting snippets are cut. This ensures the correct handling of mesh cells that are cut by multiple level sets.

#### 2.1.2. Source model and transfer matrix

Following the principle of St. Venant, the source term *f* will be approximated by a set of monopoles. Where fitted FEM use mesh vertices as monopole locations, this is not feasible for CutFEM as fundamental cells may have vertices not belonging to the source compartment. Only gray matter cut cells are used, and the locations are based on a Gauss-Legendre quadrature rule. For more information on the Venant source model, see Buchner et al. (1997) and Medani et al. (2015).

For an accurate source analysis, it is necessary to compute the EEG-forward solution for a large number, i.e., tens of thousands, of possible sources. However, the electric potential induced by a source is only of interest at a set of predetermined points, namely, the electrodes at the scalp. However, rather than solving (11) for each source individually, a transfer matrix approach (Gençer and Acar, 2004; Wolters et al., 2004) is employed, significantly reducing the amount of computation time needed.

### 2.2. Numerical validation

#### 2.2.1. Head models

For numerical evaluations, three progressively more realistic scenarios were created, two sphere models, one of which contains realistic brain tissues, and a five compartment model created from anatomical data. For each model, we will compare CutFEM and a geometry-adapted hexahedral CG-FEM approach (*Hex*) with a node shift for the geometry-adaptation of 0.33 (Wolters et al., 2007). In the first model, the UDG approach of Nüßing et al. (2016) will also be added to the comparisons. To balance computational load, *Hex* will use 1 mm meshes, whereas for CutFEM and UDG, we use a 2 mm background mesh. Additionally, in the sphere model, the convergence rate for CutFEM will be investigated by comparing models with 16, 8, 4, and 2 mm resolution. The realistic 5-compartment model will feature an additional tetrahedral head model.

##### 2.2.1.1. Shifted spheres

The first scenario contains the four spherical compartments, such as the brain, CSF, skull, and scalp. The brain sphere will be shifted to one side, simulating a situation where the subject lies down and the brain sinks to the back of the skull. Conductivities were chosen according to study by McCann et al. (2019), with the exception that the CSF and brain use the same conductivity. In terms of volume conduction, the model is thus indistinguishable from a 3-layer concentric sphere model, and analytical solutions (De Munck and Peters, 1993) can be used as benchmark. These would not be available if a realistic CSF conductivity was used. Conductivity values and radii of the compartments are shown in Table 1. Notably, the spherical geometries used here cannot properly represent the shape of the human head. They are commonly used as an initial validation in a simplified scenario where exact reference solutions are available (Wolters et al., 2004; Medani et al., 2015). Thus, they are merely the first of three numerical validation steps in this study.

**Table 1:** Radii, center, and conductivities for the shifted sphere model.

	**Radius [mm]**	**Center [mm]**	**σ [S/m]**
Scalp	92	(127 127 127)	0.43
Skull	86	(127 127 127)	0.01
CSF	80	(127 127 127)	0.33
Brain	78	(129 127 127)	0.33

TPMC was applied twice, once on the fundamental mesh and once on the resulting cut cells. Notably, this additional refinement step does not change the number of trial functions of the model. In total, 200 evenly spaced electrodes were placed on the surface of the outer layer, and a total of 13,000 Evaluation points were distributed evenly throughout the inner sphere. Lead fields for both radial and tangential source directions were computed at each point. For CutFEM, a combination of γ = 16 and γ_*G*_ = 0.1 has shown promising results. For UDG, no ghost penalty was implemented and γ = 4 was chosen, following Nüßing et al. (2016).

##### 2.2.1.2. Spheres containing realistic brain

In the previous section, the level set functions could be computed analytically up to an arbitrary accuracy. In a realistic scenario where the segmentation quality is limited by the MRI resolution as well as partial volume effects and MRI artifacts, this is not the case. An easy way to pass level sets to CutFEM is by using tissue probability map (TPM), a typical intermediate result (Ashburner et al., 2014) from segmentation which provides for each voxel the probability that it is located in a certain compartment.

To examine the performance of CutFEM when used together with TPM's, another sphere model is employed, this time containing realistic gray and white matter compartments obtained from MRI scans of a human brain. The subject was a healthy 24-year-old male from whom T1- and T2-weighted MRI scans were acquired using a 3 Tesla MRI Scanner (MagnetomTrio, Siemens, Munich, Germany) with a 32-channel head coil. For the T1, a fast gradient-echo pulse sequence (TFE) using water selective excitation to avoid fat shift (TR/TE/FW = 2300/3.51 ms/8°, inversion pre-pulse with TI = 1.1 s, cubic voxels of 1 mm edge length) was used. For the T2, a turbo spin echo pulse sequence (TR/TE/FA = 3200/408 ms/90°, cubic voxels, 1 mm edge length) was used. TPM's were extracted from both T1- and T2-MRI using SPM12 (Ashburner et al., 2014) as integrated into fieldtrip (Oostenveld et al., 2011). For each voxel, the average of both TPM's was computed, and a threshold probability of 0.4 was set as zero-line.

The inner skull surface was defined as the minimal sphere containing the entire segmented brain with CSF filling the gaps. The spherical skull and scalp were chosen to have a thickness of 6 mm. The same conductivities as before were used with CSF, and gray and white matter being identical, and again 200 sensors were placed on the scalp surface.

##### 2.2.1.3. Realistic 5 compartment head model

As an extension of the previous model, realistic 5-compartment head models were created using the same anatomical data, replacing the spherical skin, skull, and CSF by realistic segmentations. Again, level sets were created from probability maps. To obtain smooth skull and scalp surfaces in the TPM case, binary maps of the skull and skin were created following the procedure in the study by Antonakakis et al. (2020). The level sets of the skull/skin were then calculated as an average of the binary map and the T1/T2 TPM again with a threshold of 0.4. Following the study by Antonakakis et al. (2020), the level sets were cut off below the neck to reduce computational load while maintaining a realistic current flow below the skull. Again, lead fields from a hexahedral mesh were created for comparison as well as a 5-compartment tetrahedral model with surfaces created using SIMNIBS' headreco pipeline (Saturnino et al., 2019). SIMNIBS provides an automated segmentation and meshing pipeline taking both T1 and T2 MRI into account, similar to the model using TPM. Level sets were created from the surfaces, and another CutFEM model was created from these, yielding four lead fields: TPM-CutFEM and *Hex*, which are based on the tissue probability maps as well as Tri-CutFEM and*Tet*, which are based on the headreco surface triangulations. DOF, number of cut cells/mesh elements and the resulting number of snippets are shown in Table 2. Now, we have lead fields based on two different segmentation routines. TPM is closer to the original MRI while surface triangulations yield smoother surfaces at the cost of demanding nested compartments. The question which of the two segmentation routines is preferable is beyond the *n* = 1 study performed in this paper. Thus, neither method can be used as a reference solution. It is rather our goal to test CutFEM in both scenarios and showcase differences compared with the respective alternative, a standard first order tetrahedral or hexahedral FEM.

**Table 2:** Number of degrees of freedom/snippets/cut cells for CutFEM and number of degrees of freedom/elements for hexahedral/tetrahedral mesh.

	**DOF**	**Cut cells/ elements**	**Snippets**
TPM-CutFEM	917,463	716,994	7,950,120
TRI-CutFEM	1,159,831	911,567	7,647,088
*Hex*	3,909,303	3,475,138	-
*Tet*	1,135,379	6,475,318	-

#### 2.2.2. Forward and inverse comparisons

For the two spherical scenarios, analytical forward solutions were calculated as a reference. For the realistic cases, somatosensory evoked potentials were recorded, and a dipole scan was performed as described in detail in Section 2.2.2.2.

The two latter scenarios including realistic gray/white matter use a regular 2 mm source grid created using Simbio https://www.mrt.uni-jena.de/simbio/. It was ensured that the sources are located inside the gray matter compartment for both approaches (*Hex* + CutFEM). The resulting source space contains 58.542 different dipole locations with no orientation constraint being applied.

##### 2.2.2.1. Error measures

Two different metrics were employed to quantify the observed errors, the relative difference measure (RDM) and the magnitude error (MAG) (Wolters et al., 2007).

The RDM measures the difference in potential distribution at the scalp electrodes.


(12) $$RDM(\%)(u^{ana},u^{num})=50*||\frac{u^{ana}}{||u^{ana}|{|}_{2}}-\frac{u^{num}}{||u^{num}|{|}_{2}}|{|}_{2}.$$

It ranges from 0 to 100, the optimal value being 0. MAG determines the differences in signal strength at the electrodes.


(13) $$MAG(u^{ana},u^{num})=100*(\frac{||u^{num}|{|}_{2}}{||u^{ana}|{|}_{2}}-1).$$

Measured in percent, its optimal value is 0. It is unbounded from above and bound by −100 from below. *u*^*ana*^, *u*^*num*^∈ℝ^*s*^ contain the analytical and numerical potential at the *s* different sensor locations.

CutFEM is implemented into the DUNEuro toolbox https://www.medizin.uni-muenster.de/duneuro (Schrader et al., 2021), where the FEM calculations were performed. Analytical EEG solutions were calculated using the fieldtrip toolbox (Oostenveld et al., 2011). An example data set including somatosensory data was uploaded to Zenodo https://zenodo.org/record/3888381#.Yf0tT_so9H4.

For a comparison of runtime and memory usage, the forward calculation is split into five steps. The time necessary to create a driver, i.e., the time DUNEuro needs to setup the volume conductor, the times needed to assemble the stiffness matrix and AMG solver, the transfer matrix solving process using Dune-ISTL (Bastian et al., 2021), and the calculation of the final lead field matrix. All computations are performed on a bluechip workstation with an AMD Ryzen Threadripper 3960X and 128 GB RAM. A total of 16 threads are used to calculate the 200 transfer matrix/lead field columns in parallel. In the current implementation, CutFEM is limited to six compartments but that is an arbitrary restriction which can be increased at will.

##### 2.2.2.2. Somatosensory data and dipole scan

To investigate CutFEM's influence on source reconstruction, an electric stimulation of the median nerve was performed on the same subject the anatomical data was acquired from. The subject gave written informed consent before the experiment and had no history of neurological or psychiatric disorders. The institution's ethical review board (Ethik Kommission der Ärztekammer Westfalen-Lippe und der WWU) approved all experimental procedures on 2 February 2018 (Ref. No. 2014-156-f-S). The stimuli were monophasic square-wave pulses of 0.5ms width in random intervals between 350 and 450ms. The stimulus strength was adjusted such that the right thumb moved clearly. EEG data were measured using an 80 channel cap (EASYCAP GmbH, Herrsching, Germany, 74 channel EEG plus additional 6 channels EOG to detect eye artifacts). EEG positions were digitized using a Polhemus device (FASTRAK, Polhemus Incorporated, Colchester, Vermont, U.S.A.). In total, 2,200 stimuli were digitally filtered between 20 and 250 Hz (50 Hz notch) and averaged to improve signal-to-noise ratio. A single dipole scan was conducted over the whole source space using the data at the peak and the CutFEM lead field.

The P20/N20 component typically exerts a high signal-to-noise ratio and a strongly dipolar topography, making it an ideal candidate for a dipole scan approach as motivated for example by Buchner et al. (1994).

## 3. Results

### 3.1. Shifted sphere model

**Figure 2:**
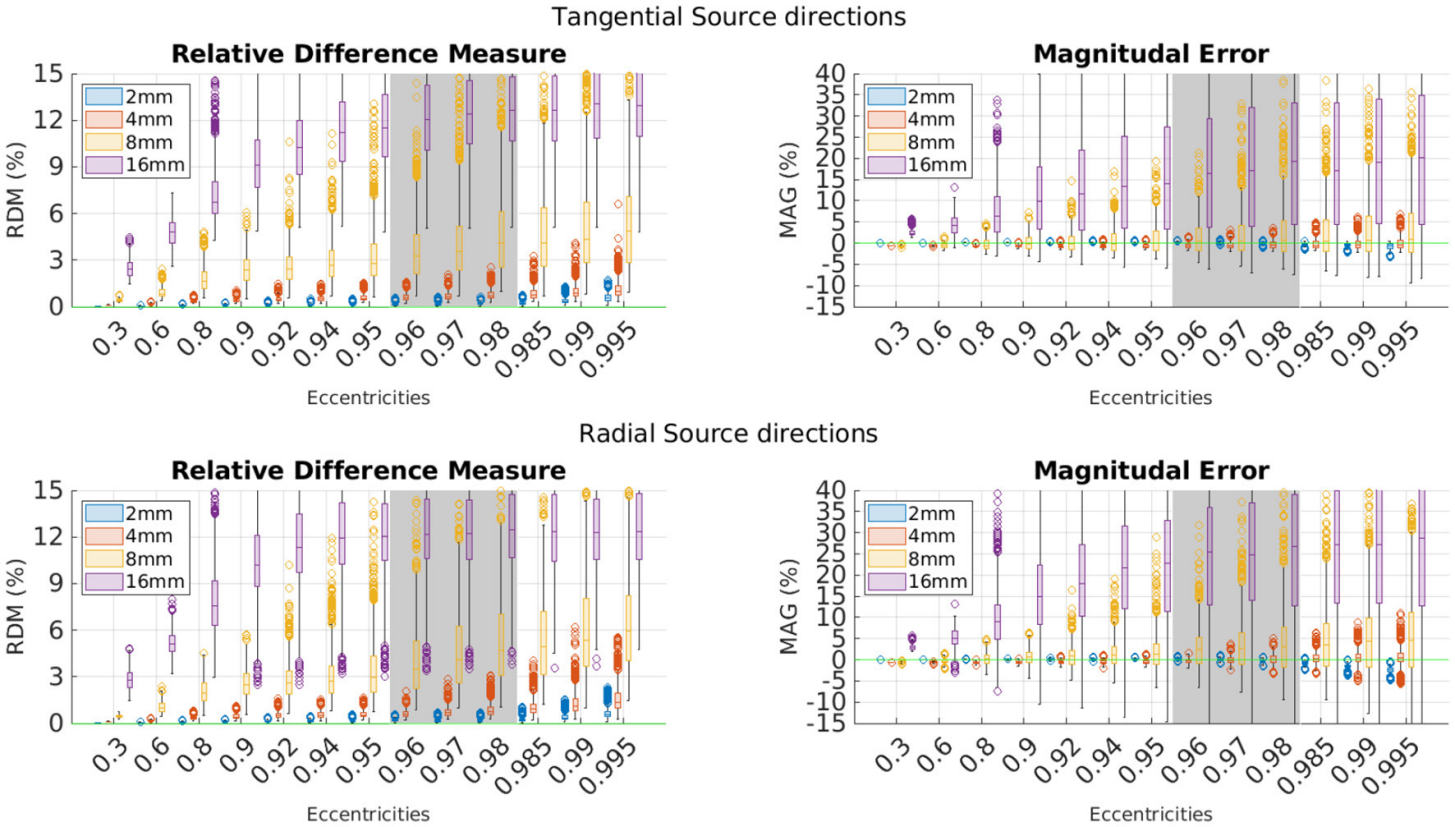
EEG forward modeling errors for different fundamental mesh resolutions in a shifted sphere scenario. **Top**: Errors for tangential source directions. **Bottom**: Errors for radial source directions. Errors are in percent and grouped by eccentricities. The green line marks optimal error values. The gray area indicates the physiologically most realistic eccentricities.

The first investigated model is the shifted sphere scenario, where the brain sphere was moved within the CSF-sphere until there was exactly one contact point between the skull and brain (see 2.2.1.1). In Figure 2, the convergence speed for both radial and tangential source directions can be seen. Fundamental meshes with a resolution of 16, 8, 4, and 2 mm were created yielding finite element spaces with 4600/21401/111,192 and 552,985 DOF, respectively. Mean RDM decreases from 10.54 to 3.47 to 0.63 to 0.18 while the mean of the absolute value of the MAG decreases from 17.63 to 3.37 to 0.80 to 0.33. A 2 mm resolution, thus, already yields excellent numerical results.

When comparing number of DOF and RAM usage, it is clear that CutFEM is by far the most memory efficient approach, using approximately one-fifth of the number of trial functions and approximately one-tenth of the amount of RAM as UDG (Table 3). *Hex* also uses significantly more resources than CutFEM.

**Table 3:** Computation times, RAM/degree of freedom usage in the shifted sphere model.

	**CutFEM**	**UDG**	***Hex***
Number DOF	552 985	3 601 824	3 341 280
Max. RAM used	6.91 GB	64.77 GB	40.2 GB
Driver setup	44 s	45 s	52 s
Matrix assembly	319 s	161 s	25 s
Solver setup	353 s	235 s	45 s
Solving	1,111 s	2,367 s	1,550 s
Lead field	22 s	20 s	125 s
Total time	1,849 s	2,828 s	1,797 s

Regarding computation time, as UDG has to solve a significantly larger system, each iteration step in the solution phase takes longer than for CutFEM. As most time is spent on solving the system, CutFEM is overall approximately 16 min or 34% faster than UDG. The same cannot be said for comparisons to the standard *Hex* approach. While each iteration of the solver required less time than for *Hex*, it required an average of 92 iterations compared with 14 for *Hex*. The unfitted approaches spend less time calculating the final lead field as the time needed to locate each dipole within the 2 mm background mesh is lower than the 1 mm hexahedral mesh. In total, the hexahedral CG was only faster than CutFEM by a negligible 3% or 52 s.

**Figure 3:**
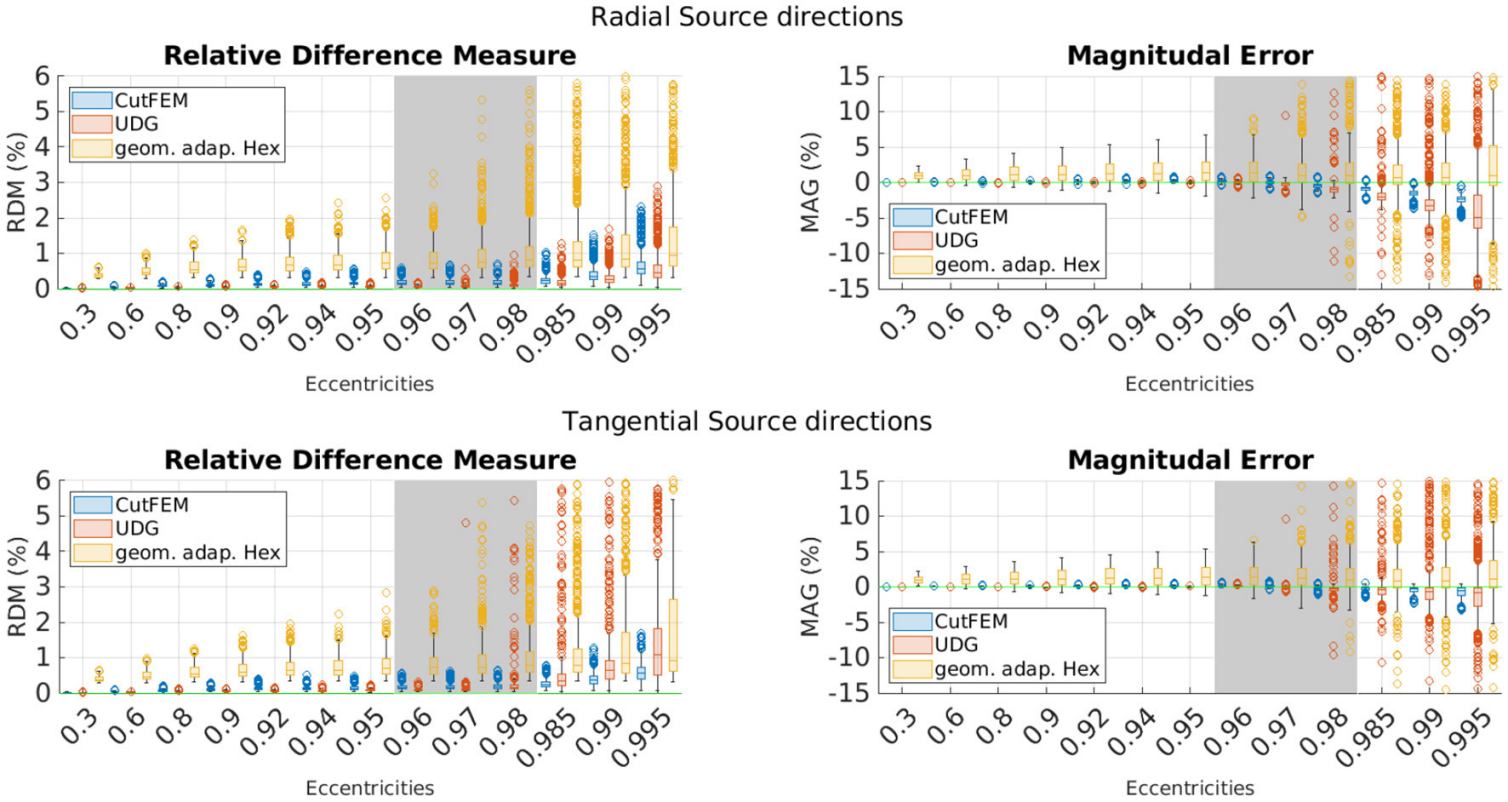
EEG forward modeling errors for *Hex* and unfitted FEM approaches in a shifted sphere scenario. **Top**: Errors for tangential source directions. **Bottom**: Errors for radial source directions. Errors are in percent and grouped by eccentricities. The green line marks optimal error values. The gray area indicates the physiologically most realistic eccentricities.

Error comparisons between CutFEM, UDG, and *Hex* are shown in Figure 3. CutFEM outperforms *Hex* in all eccentricity categories and for both radial and tangential source directions. As the pyramidal cells that give rise to the EEG potential are located in layer 5 of the gray matter (Murakami and Okada, 2006), eccentricities corresponding to 1–2 mm distance to the skull are the physiologically most relevant. For eccentricities between 0.96 and 0.98 and both source directions, CutFEM has average RDM/MAG values of 0.18 and −0.06%, comparable to UDGs 0.17 and −0.2% and significantly lower than *Hex*'s 0.94 and 1.57%.

The most pronounced differences are at low eccentricities or when looking at magnitudes. CutFEM performance is similar for both radial and tangential source directions, and UDG shows similar or slightly better results at low eccentricities. However, except for radial RDM's, UDG deteriorates faster at high eccentricities above 0.98. As both operate on the same cut mesh, the larger variance in the UDG results can most likely be explained by CutFEM's use of the ghost penalty stabilization. The overall largest absolute error values for CutFEM are 3.08 % RDM and 8.21 % MAG, underlining its performance with regard to outliers. Due to the similar numerical accuracy of CutFEM and UDG, we will only compare CutFEM and *Hex* in the following scenarios.

### 3.2. Sphere containing realistic brain

The results in the previous section were achieved using analytically computed level sets. Deviating from this, we will now use a semi-realistic case where realistic brain compartments are contained within spheres. Again, several different penalty parameters were tried, showing that a combination of γ = 40 and a ghost penalty of γ_*g*_ = 0.5 yield good results for CutFEM.

**Figure 4:**
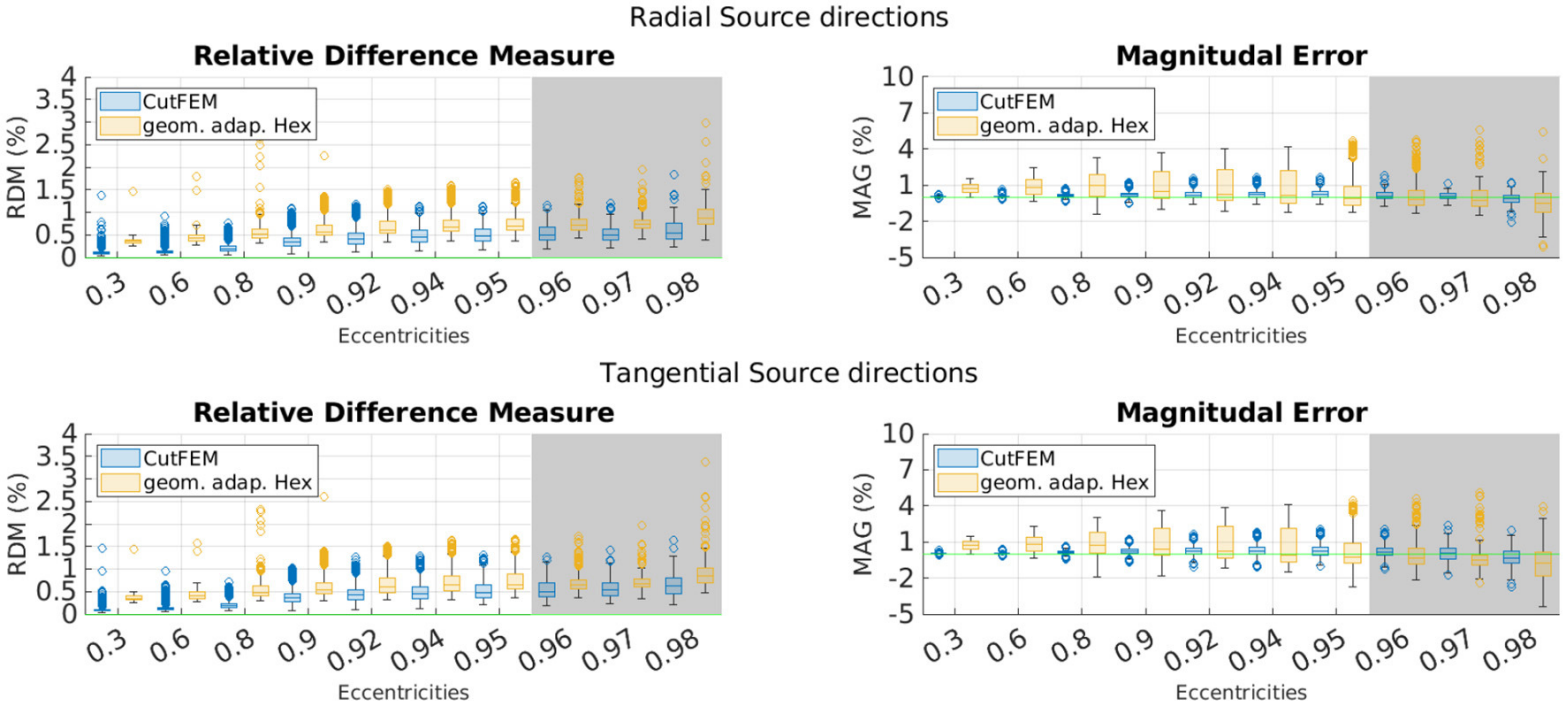
Overview of different EEG-errors for five layer continuous Galerkin- and CutFEM approaches using realistic brain compartments contained in spherical skull and scalp shells. **Top**: Errors for tangential source directions. **Bottom**: Errors for radial source directions. Errors are in percent and grouped by eccentricities. The green line marks optimal error values. The gray area indicates the physiologically most realistic eccentricities.

The results are presented in Figure 4. Notably, eccentricity is stated with respect to the distance to the skull. As source points are only inside the gray matter, the number of source points at high eccentricities is much lower. The eccentricity groups 0.98, 0.985, 0.99, and 0.995 were thus combined into one group containing 136 points.

Much like before, CutFEM remains well below 1.5 and 2% RDM and MAG, respectively, whereas *Hex* has higher median values for nearly all eccentricities and more outliers going up to more than 1.5% RDM and 4% MAG. CutFEM is again more stable with regard to outliers and especially when looking at magnitudes, differences between the two methods are in the several percent range.

Overall, it can be stated that CutFEM is about as fast as and more accurate than *Hex* and about as accurate as and faster than UDG.

### 3.3. Realistic 5-compartment head model

For the final part of this study, two lead fields, one from CutFEM, one from hexahedral CG, were created using realistic 5-compartment head models including the gray and white matter, CSF, skull and scalp tissues. Somatosensory evoked potentials were acquired from a medianus stimulation of the right hand.

#### 3.3.1. Lead field differences

Before looking at inverse reconstructions, we will investigate the differences between the forward results. As the same source space and electrodes were used for both models, we can again compute MAG and RDM values. In the absence of an analytical solution, these measurements cannot capture errors but rather differences between the methods without making a clear statement which is more accurate.

**Figure 5:**
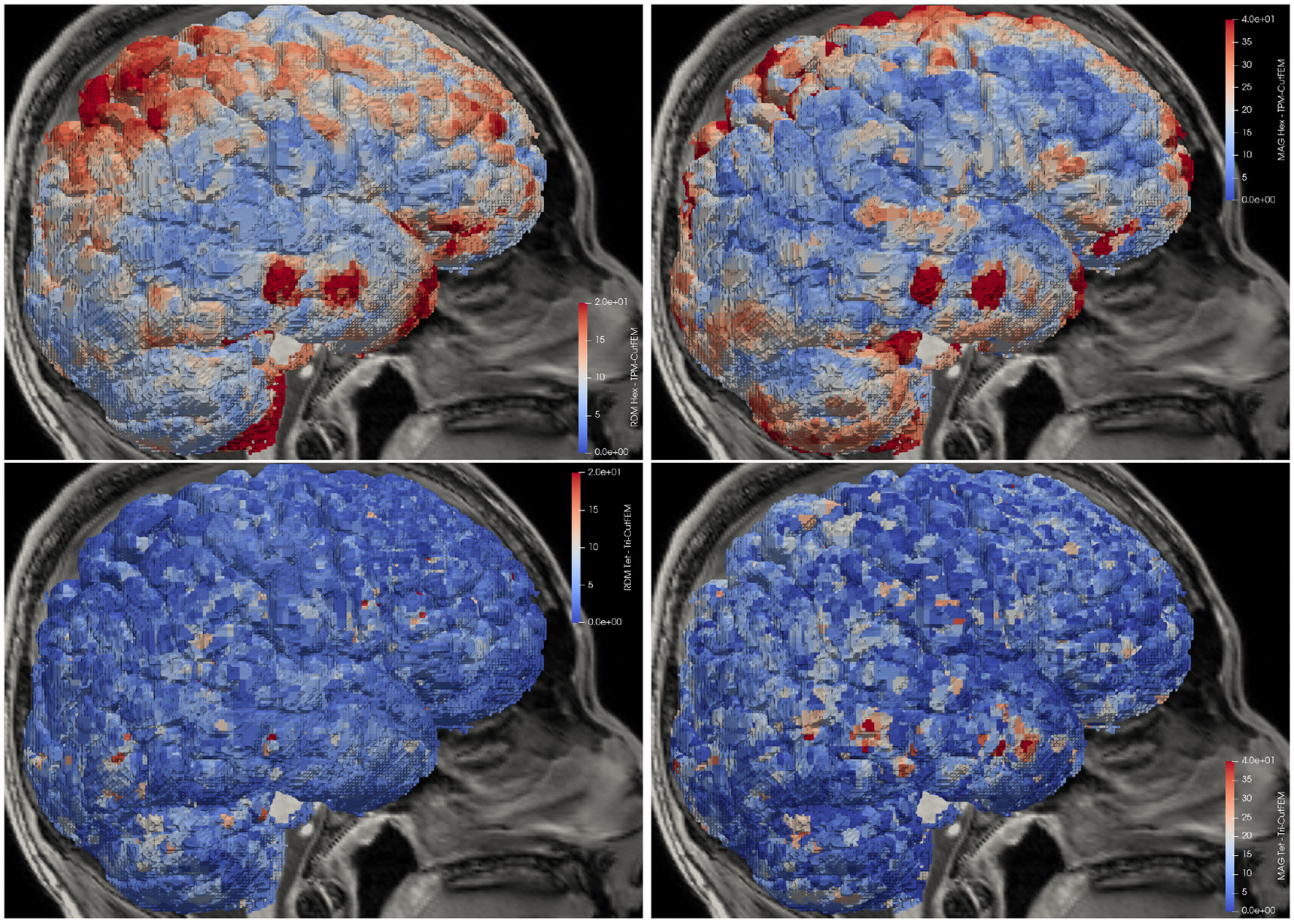
Lead field differences in distribution and magnitude. **Top**: TPM-CutFEM vs. *Hex*, **Bottom**: Tri-CutFEM vs. *Tet*. Differences are interpolated onto the gray matter.

For visualization purposes, for each gray matter centerpoint of the *Hex* mesh, the closest source point is identified, RDM and MAG are computed for each spatial direction and averages over the directions are calculated. The results are shown in Figure 5. Looking first at the differences between the *Hex* and TPM-CutFEM model, we see that in both measures, the highest differences can be observed in inferior areas near the foramen magnum and optic channels or in superior areas. Overall, the difference in potential distribution was 9.40 ± 4.15% and the difference in magnitude was 18.94 ± 12.03%. Interestingly, with a correlation coefficient of only 0.22, high RDM values do not necessarily coincide with high MAG values.

When comparing *Tet* to Tri-CutFEM we see that the differences are significantly smaller. With RDMs of 4.59 ± 3.54% and MAGs of 8.74 ± 8.30%, they average less than half the differences between the TPM-based models. Additionally, the differences between Tri-CutFEM and TPM-CutFEM are 4.52± 2.86% (RDM)/0.03 ± 14.50%(MAG) lower than when comparing *Tet* and *Hex*. This is to be expected as the CutFEM lead fields only differ in the way the surfaces are provided while the differences between hexahedral and tetrahedral FEM also encompass geometry adaptation, multi-linear vs. linear FE-spaces and local differences in mesh resolution.

#### 3.3.2. Reconstruction of somatosensory stimulation

**Figure 6:**
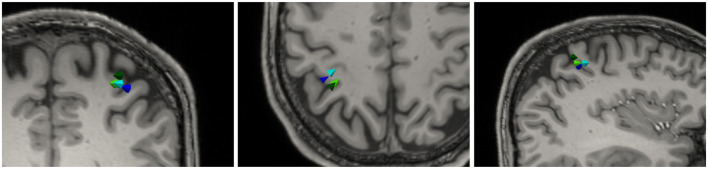
Dipole reconstruction results of P20 component of the medianus stimulation based on four different lead fields: *Tet* (dark green), *Hex* (dark blue), Tri-CutFEM (light blue), and TPM-CutFEM (light green). **Left** to **right**: Axial, Coronal, and Sagittal view.

Finally, all four lead fields were used to perform a source reconstruction of the P20 component of an electric wrist stimulation. Dipole scans were conducted over the entire source space, the results of which are shown in Figure 6. In total, 93.03 and 92.15% of the data could be explained the TPM-CutFEM and the *Hex* lead field, respectively, resulting in dipole strengths of 5.8 and 7.56 nAm. These are slightly weaker than the Tri-CutFEM and *Tet* dipoles at 8.1 and 8.7 nAm, respectively. From the literature (Buchner et al., 1994), one expects the P20 component to be located in Brodmann Area 3b, located in the anterior wall of the postcentral gyrus (and oriented toward the motor cortex). This is in line with the TPM-CutFEM reconstruction while the other three lead fields yield reconstructed dipoles that located on the posterior wall. Overall, the CutFEM-based reconstructions are located slightly more medial and frontal than their counterparts. While this is only a single subject study, it shows that the choice of the FEM method can significantly change the localization result of a dipole scan.

## 4. Discussion

The purpose of this study is to introduce CutFEM, an unfitted FEM for applications in EEG forward modeling. After discussing the mathematical theory behind CutFEM and implementational aspects, three progressively more realistic scenarios are introduced, ranging from a multi-layer sphere model to the reconstruction of somatosensory evoked potentials.

At similar computation times, CutFEM shows preferable results when compared with a geometry-adapted hexahedral CG-FEM (Wolters et al., 2007) in both a shifted sphere scenario and a sphere model with realistic brain tissues. While CutFEM requires significantly less DOF, both methods require similar computation times due to the different number of solver iterations. Thus, a thorough investigation of different iterative solver techniques such as multigrid methods and possibly a modification of the ghost penalty will be a part of future studies.

Compared with UDG (Bastian and Engwer, 2009), it is shown that CutFEM combined with a ghost penalty leads to a decrease in outlier values at high eccentricities as well as a significant reduction in memory consumption and computation time.

Using a realistic five-compartment head model based on either tissue probability maps or surface triangulations, we found larger differences compared with standard hexahedral or tetrahedral first order FEM when using TPM. Of all four computed lead fields, only CutFEM in conjunction with tissue probability maps correctly localizes the somatosensory P20 in the expected Brodmann area 3b. Especially in applications such as presurgical epilepsy diagnosis, such accurate reconstructions might contribute significantly to the correct localization of the irritative zone (Neugebauer et al., 2022). The employed somatosensory experiment featured clear peaks and a high signal-to-noise ratio, making it an ideal candidate for an initial study. Further investigations and a larger study size are necessary to determine CutFEM's contribution to accurate source reconstructions when used with noisier data and/or more advanced inverse methods.

In the study by Vallaghé and Papadopoulo (2010), a trilinear immersed FEM approach was introduced that like CutFEM employs level sets as tissue surfaces. Rather than using a Nitsche-based coupling, continuity of the electric potential is enforced by modifying the trial function space. Compared with CutFEM, no free parameters such as γ and γ_*G*_ are introduced but the absence of overlapping submeshes means that there is no increased resolution in areas with complex geometries.

In the study by Windhoff et al. (2013), Nielsen et al. (2018), the process of building a tetrahedral mesh from segmentation data is investigated. Surface triangulations that are free of topological defects, self-intersections, or degenerate angles have to be created before volumetric meshing. The authors show that it is possible to create such high quality surfaces and subsequent tetrahedral meshes for realistic head models; however, they may come at the cost of modeling inaccuracies such as the separation of the gray matter and skull by a thin layer of CSF.

A main advantage of CutFEM is its flexibility with regard to the anatomical input data. Level sets can be created from a variety of sources, such as tissue probability maps, binary images, or surface triangulations. This simplifies the question of how to create a mesh from segmentation data. However, CutFEM does not answer the question which of these sources should be used in future. Numerically, one can expect the smoother level sets created from surface triangulations to produce fewer distorted cut cells than those created from TPM. As shown in the results though, CutFEM is stable with regard to tissue probability maps. Future investigations will show whether staying close to the raw MRI data by using tissue probability maps is preferable over having nested, smooth surfaces as required for tetrahedral models. The *n* = 1 study we performed here cannot conclusively answer this question. From an anatomical perspective, CutFEM now offers the possibility to accurately model supine subject positioning where the brain touches the skull. Quantifying the impact, this has on EEG source estimation will also be a part of future investigations.

## 5. Conclusion

CutFEM performed well both when the underlying head model was created using analytical level sets or realistic segmentation results. Application to an inverse reconstruction of a somatosensory evoked potential yielded findings that are in line with the literature. The level sets underlying CutFEM impose few restrictions on the compartments, thus allowing for more simplified segmentation routines when compared with other FEM approaches using surface triangulations.

## Data availability statement

The data analyzed in this study is subject to the following licenses/restrictions: the software in which the new methodology was implemented can be found under https://www.medizin.uni-muenster.de/duneuro, an example data set can be found under https://zenodo.org/record/3888381#.Yf0tT_so9H4. The dataset used in the realistic head model section is from a different subject than the one uploaded to Zenodo but otherwise identical. Requests to access these dataset should be directed to tim.erdbruegger@uni-muenster.de.

## Ethics statement

The studies involving human participants were reviewed and approved by Ethik Kommission der Ärztekammer Westfalen-Lippe und der WWU (Ref. No. 2014-156-f-S). The patients/participants provided their written informed consent to participate in this study.

## Author contributions

CE, AW, and CW: conceptualization. CE, TE, AW, and CW: methodology. CE, TE, and AW: software. TE: investigation and writing—original draft. YB, CE, TE, JG, MH, RL, J-OR, and CW: writing—reviewing and editing. CE and CW: supervision. AB, JG, RL, SP, FW, and CW: funding acquisition. All authors contributed to the article and approved the submitted version.
